# You Look Human, But Act Like a Machine: Agent Appearance and Behavior Modulate Different Aspects of Human–Robot Interaction

**DOI:** 10.3389/fpsyg.2017.01393

**Published:** 2017-08-23

**Authors:** Abdulaziz Abubshait, Eva Wiese

**Affiliations:** Department of Psychology, George Mason University, Fairfax VA, United States

**Keywords:** mind perception, social robotics, intentionality, human–robot interaction, social cognition

## Abstract

Gaze following occurs automatically in social interactions, but the degree to which gaze is followed depends on whether an agent is perceived to have a mind, making its behavior socially more relevant for the interaction. Mind perception also modulates the attitudes we have toward others, and determines the degree of empathy, prosociality, and morality invested in social interactions. Seeing mind in others is not exclusive to human agents, but mind can also be ascribed to non-human agents like robots, as long as their appearance and/or behavior allows them to be perceived as intentional beings. Previous studies have shown that human appearance and reliable behavior induce mind perception to robot agents, and positively affect attitudes and performance in human–robot interaction. What has not been investigated so far is whether different triggers of mind perception have an independent or interactive effect on attitudes and performance in human–robot interaction. We examine this question by manipulating agent appearance (human vs. robot) and behavior (reliable vs. random) within the same paradigm and examine how congruent (human/reliable vs. robot/random) versus incongruent (human/random vs. robot/reliable) combinations of these triggers affect performance (i.e., gaze following) and attitudes (i.e., agent ratings) in human–robot interaction. The results show that both appearance and behavior affect human–robot interaction but that the two triggers seem to operate in isolation, with appearance more strongly impacting attitudes, and behavior more strongly affecting performance. The implications of these findings for human–robot interaction are discussed.

## Introduction

In social interactions, we use information from gestures, facial expression or gaze direction to make inferences about what others think, feel or intend to do (i.e., *mentalizing*; Adolphs, 1999; Emery, 2000; Gallagher and Frith, 2003). How we react to these cues is determined by how much social relevance we ascribe to them and, specifically, to what degree they are believed to originate from an entity with a mind, capable of having internal states like emotions or intentions (i.e., *mind perception*; Gray et al., 2007). Changes in gaze direction, for instance, are followed more strongly when they are displayed by a face with a fearful rather than a neutral expression (Graham et al., 2010), or when they are believed to be intentional rather than pre-programmed or random (Teufel et al., 2009; Wiese et al., 2012; Wykowska et al., 2014; Özdem et al., 2016). Seeing minds in others is not exclusive to human agents, but intentionality can also be ascribed to agents who do not have minds (i.e., robots) or whose mind status is ambiguous (i.e., animals; Gray et al., 2007).

In order to trigger mind perception, non-human entities need to display signs of intentionality via appearance (Kiesler et al., 2008; Looser and Wheatley, 2010; Admoni et al., 2011) and/or behavior (Morewedge, 2009; Waytz et al., 2010; Wiese et al., 2014). Entities that physically resemble humans are more likely to be perceived as ‘having a mind’ than agents with a mechanistic appearance, in particular when they display human facial features (DiSalvo et al., 2002; Kiesler et al., 2008; Tung, 2011). Entities without human appearance can still trigger mind perception when their behavior is predictable (Morewedge, 2009; Pfeiffer et al., 2011), leads to negative outcomes (Waytz et al., 2010), or resembles movement patterns reminiscent of human–human interactions (Heider and Simmel, 1944; Abell et al., 2000; Castelli et al., 2000). Behavior is also interpreted as intentional when it is believed to be reliable (Süßenbach and Schönbrodt, 2014; Wiese et al., 2014) or to be generated by a human (Wiese et al., 2012; Wykowska et al., 2014; Özdem et al., 2016).

A positive effect of mind perception on attitudes and performance has also been observed in human–robot interaction (Sidner et al., 2004; Bennewitz et al., 2005; Mutlu et al., 2006, 2012; Fussell et al., 2008; Yamazaki et al., 2010; Huang and Thomaz, 2011; Staudte and Crocker, 2011; Pfeiffer-Lessmann et al., 2012). Robots that exhibit human gestures like shrugging or nodding, for instance, have a positive impact on emotional reactions and perceived trustworthiness (Kiesler et al., 2008; Carter et al., 2014), and robots displaying human behavior lead to improved performance on joint tasks (Breazeal et al., 2005; Looije et al., 2010; Pak et al., 2012; Waytz et al., 2014). In contrast, robots that do not trigger mind perception have negative effects on performance in social interactions (Wiese et al., 2012; Wykowska et al., 2014; Caruana et al., 2016; Özdem et al., 2016), and fail to induce social facilitation effects (Bartneck, 2003; Woods et al., 2005; Park and Catrambone, 2007; Riether et al., 2012).

While these studies suggest that mind perception in non-human agents (a) has a beneficial effect on attitudes and performance in human–robot interaction, and (b) can be triggered experimentally via appearance and/or behavior, no study to date has examined how congruent (i.e., cue A and B both trigger or inhibit mind perception) versus incongruent (i.e., cue A/B triggers/inhibits mind perception) combinations of these triggers affect attitudes and performance in human–robot interaction. The current study addresses this question by manipulating the likelihood that mind is ascribed to non-human agents via appearance (high: human-like vs. low: robot-like) and behavior (high: predictable vs. low: random), and examining how congruent (human-like/reliable, robot-like/random) versus incongruent (human-like/random, robot-like/reliable) combinations of these triggers affect gaze following (i.e., performance measure) and agent ratings (i.e., attitude measure). Gaze following was picked as performance measure in the present experiment, since gaze direction is one of the most important cues in social interactions indicating another’s focus of interest, and a pre-requisite for more complex social-cognitive functions like mentalizing (Baron-Cohen, 1995; Frith and Frith, 2006).

When examining gaze following experimentally, a face is presented centrally on the screen that first gazes straight ahead, and then changes gaze direction to trigger shifts of the observer’s attention to the left or right side of the screen (i.e., *gaze cueing*; Friesen and Kingstone, 1998). This gaze cue is followed by the presentation of a target either at the cued location (i.e., valid trial) or an uncued location (i.e., invalid trial), with reactions to targets appearing at the cued location being faster than reactions to targets appearing at an uncued location (*gaze-cueing effect*; Friesen and Kingstone, 1998; Frischen et al., 2007). Positive effects of gaze cues have also been observed in human–robot interaction, where robots that shift their gaze during social interactions are perceived as more enjoyable than robots that do not shift their gaze (Kanda et al., 2001), and robots that conjointly attend to where the human partner is looking are perceived as more competent than robots that do not engage in joint attention (Huang and Thomaz, 2011). Robot gaze also helps performance on joint human–robot tasks, for instance, by improving the accuracy of predictions in an object selection game (Mutlu et al., 2009), or by improving recollection in a memory task by gazing at relevant objects (Mutlu et al., 2006).

Attentional orienting to gaze cues has traditionally been thought of as a bottom–up process that is observable in infants as young as 3 months of age (Hood et al., 1998), and can be triggered by any kind of stimulus with eye-like configurations (Friesen and Kingstone, 1998; Langton and Bruce, 1999; Quadflieg et al., 2004). Confirming its reflexive nature, gaze following cannot be suppressed even when gaze direction is unlikely to predict the location of a target (Friesen et al., 2004; Vecera and Rizzo, 2006), and is not modulated by the gazer’s *animacy* (Quadflieg et al., 2004), *familiarity* (Frischen and Tipper, 2004), *facial expression* (Hietanen and Leppänen, 2003; Bayliss et al., 2007), or *trustworthiness* (Bayliss and Tipper, 2006). The few modulatory effects that were originally reported in the context of gaze following strongly depended on age (i.e., stronger gaze following in children; Hori et al., 2005), and individual traits (i.e., stronger gaze cueing in highly anxious individuals; Tipples, 2006; Fox et al., 2007).

More recently, however, studies have shown that gaze following *can* be top–down modulated when gaze behavior is embedded in a context that enhances its social relevance for the observer (Tipples, 2006; Fox et al., 2007; Bonifacci et al., 2008; Graham et al., 2010; Kawai, 2011; Hungr and Hunt, 2012; Süßenbach and Schönbrodt, 2014; Wiese et al., 2014; Wykowska et al., 2014; Cazzato et al., 2015; Dalmaso et al., 2016). Using this updated version of the original gaze-cueing paradigm, researchers were able to show that variables like *similarity-to-self* (Hungr and Hunt, 2012; Porciello et al., 2014), *physical humanness* (Admoni et al., 2011; Martini et al., 2015), *facial expression* (Bonifacci et al., 2008; Graham et al., 2010), *social status* (Jones et al., 2010; Dalmaso et al., 2012, 2014, 2015, 2016; Ohlsen et al., 2013), *membership in ingroup* (Dodd et al., 2011, 2016; Liuzza et al., 2011; Pavan et al., 2011; Ciardo et al., 2014; Cazzato et al., 2015; Dalmaso et al., 2015), or *familiarity* (Frischen and Tipper, 2006; Deaner et al., 2007) are able to modulate the degree to which gaze is followed by increasing or decreasing its social relevance.

Believing that an agent is intentional rather than pre-programmed is another factor that can increase the social relevance of observed behavior, with the effect that malevolent actions believed to be intentional are experienced more intensely (Gilbert et al., 2004; Gray and Wegner, 2008), and judged more harshly (Ohtsubo, 2007; Cushman, 2008) than unintentional ones. Similarly, believing that changes in gaze direction are intentional versus unintentional increases the degree to which they are followed (Teufel et al., 2009; Wiese et al., 2012, 2014; Wykowska et al., 2014), and positively affects how the gazer is evaluated (Bayliss and Tipper, 2006). Altogether, these studies indicate that perceiving robots as agents with a mind and the ability to execute intentional actions has the potential to positively impact performance and attitudes in human–robot interaction. What is still unclear is, which agent features most effectively trigger mind perception and how attitudes and performance in human–robot interaction are affected when two triggers, like appearance and behavior, are in conflict. The effect of conflicting agent features on attitudes and performance, however, is an important issue in human–robot interaction since a subset of contemporary robots either display human appearance or intentional behavior, but usually not both (Fong et al., 2003).

**FIGURE 1:**
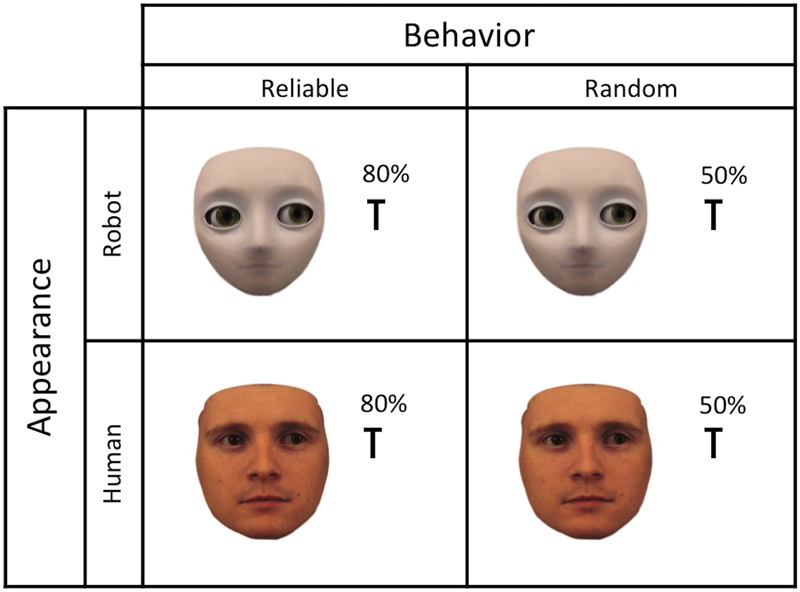
Manipulation of mind judgments: human-like appearance (80% physical humanness) and reliable behavior (80% predictive cueing) should increase the likelihood that mind is attributed, while robot-like appearance (20% physical humanness) and random behavior (50% predictive cueing) should decrease the likelihood that mind is attributed to an agent.

In the current experiment, we examine how behavior and appearance interact in triggering mind perception, and measure how social-cognitive performance (i.e., gaze following) and agent ratings (i.e., judgments of mind status) are affected in congruent versus incongruent conditions. Based on previous studies, we expected that reliable gaze behavior (i.e., cue predicts target location in 80% of trials) and human-like appearance (i.e., 80% physical humanness) would increase the likelihood that mind is perceived in artificial agents, while random gaze behavior (i.e., cue predicts target location in 50% of trials) and robot-like appearance (i.e., 20% physical humanness) were expected to decrease the likelihood for mind perception; see **Figure 1**.

## Materials and Methods

### Participants

Eighty-six undergraduate students at George Mason University were originally recruited for the experiment. The data of 23 participants had to be excluded from analysis since they did not meet the *a priori* accuracy cut off of 90%; the data of the remaining 63 participants was analyzed (47 females, *M* age: 21, *SD* = 3.3, 10 left handed). Participants were recruited using the participant management website SONA Systems at George Mason University. Participants were randomly assigned to one of the two reliability conditions (i.e., human behavior: 80% reliable vs. robot behavior: 50% reliable), with 32 participants (24 females, *M* age: 20.6, *SD* = 3.9, three left-handed) in the 80% reliability condition and 31 participants (23 females, mean age: 19.7, *SD* = 2.5, six left-handed) in the 50% reliability condition. Approval by the Internal Review Board (IRB) was obtained prior to data collection. Participant data was collected according to George Mason University’s ethics committee. All participants gave informed consent, and reported normal to corrected-to-normal vision. Participant data was stored anonymously according to IRB guidelines. Testing time was about 30 min.

### Apparatus

Stimuli were presented on a 19-inch ASUS VB Series VB198T-P monitor with the refresh rate set at 85 Hz. RT measures were based on standard keyboard responses. Participants were seated approximately 57 cm from the monitor, and the experimenter ensured that participants were centered with respect to the monitor. The experiment was programmed using the software *Experiment Builder* (SR Research, Ltd., Ottawa, ON, Canada).

### Stimuli

Images of two agents were used for the gaze-cueing task: a robot-like agent and a human-like agent. The agent images were created by morphing a human face (i.e., male face from the Karolinska Institute database; Lundqvist et al., 1998) into a robot face (i.e., Meka S2 robot head) in steps of 10% using the software Fantamorph. Out of the morphing spectrum, the morph with 80% physical humanness was used as human-like gazer and the morph with 20% physical humanness as robot-like gazer. The left-and rightward gazing faces were created using Photoshop by shifting the irises and pupils in the eyes of the original faces until they deviated 0.4° from direct gaze, which was then followed by another round of morphing for the left- and the rightward gazing faces separately. As a last step, Gimp was used for all images to touch up any minor imperfections in images and to make the sequencing of the images smooth. The face stimuli were 6.4° wide and 10.0° high on the screen, depicted on a white background and presented in full frontal orientation with eyes positioned on the central horizontal axis of the screen; see **Figure 1**.

The target stimuli for the gaze-cueing procedure were black capital letters (F or T), measuring 0.8° in width and 1.3° in height. Targets appeared on the horizontal axis, and were located 6.0° from the center of the screen. Targets appeared at the gazed-at location in 80% of the trials in the reliable condition (i.e., gaze direction predictive of target location), and in 50% of the trials in the random condition (i.e., gaze direction non-predictive of target location); see **Figure 1**.

### Procedure

At the beginning of the session, participants gave informed consent and were randomly assigned to one of two reliability conditions (80% vs. 50%). They were then told that they would perform a gaze following task together with two different agents (introduced via images), which required discriminating target letters (F or T) by pressing one of two response keys: for half of the participants, F was assigned to the “D” key and T to the “K” key of the keyboard; for the other half of the participants, stimulus-response mapping was reversed. The original key labels on the keyboard were covered with stickers to prevent letter interference effects. Participants were informed that agent gaze either validly or invalidly cued the location of the target, and were told that the experiment started with a practice block consisting of 20 trials, followed by two experimental blocks of gaze following (one per agent). They were instructed to fix their gaze on a centrally presented fixation cross at the beginning of each trial and to remain fixated until the trial was over. After the fixation cross, the image of one of the agents would appear in the center of the screen, which would then shift its gaze left- or rightward to either validly or invalidly cue the location of the target. Participants were asked to respond as quickly and accurately as possible to the identity of the target letter as soon as it appeared on the screen. In addition to gaze following, participants were also instructed to rate the agents regarding their capability of having a mind (i.e., “Do you think this agent has a mind?”) on an eight-point Likert-scale, once at the beginning and the end of each block (all instructions were given in written form). This question was used in the current experiment to be consistent with previous literature that operationalized mind perception as the degree to which agents were judged as having a mind as a function of their physical humanness (Looser and Wheatley, 2010; Hackel et al., 2014; Martini et al., 2016). Although this question has been commonly used in the literature to assess mind perception, we would like to point out that it most likely does not measure *perceptions* of mind (i.e., actually seeing mind in the agent), but more likely measures *judgments* of mind (i.e., how similar does this agent look to agents that have a mind). In consequence, ratings probably do not reflect the degree to which participants thought the depicted agents actually *have* minds, but more likely reflect how similar they thought the agents *looked* to human agents (leaving aside that they do not actually have a mind). To account for this, the results of the agent ratings will be referred to as *mind judgments*.

**FIGURE 2:**
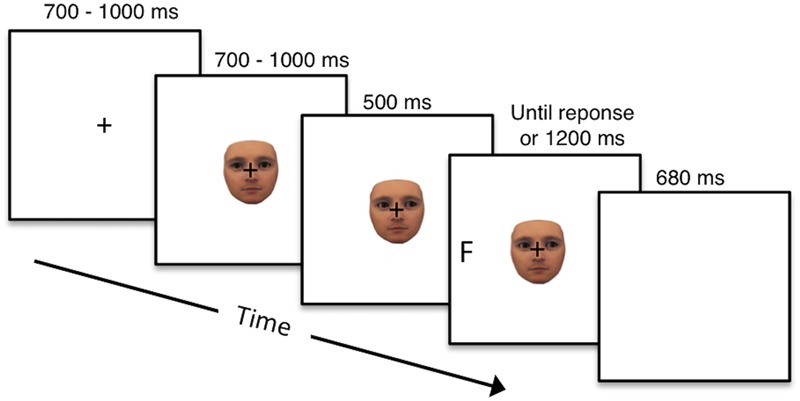
Sequence of events on a trial of gaze cueing: participants first fixated on a fixation cross for 700–1000 ms and were then presented with an agent (human vs. robot) looking straight for 700–1000 ms, followed by a change in gaze direction (either to the left or right side of the screen). After a SOA of 500 ms, the target letter (F or T) appeared either where the face was looking (valid) or opposite of where the face was looking (invalid). The target remained on the screen until a response was given or a timeout of 1200 ms was reached. A blank screen marked the end of the trial and was presented for 680 ms.

**Figure 2** illustrates the sequence of events on a given trial of gaze cueing: the trial started with the presentation of a fixation cross in the center of the screen for a random time interval of 700–1000 ms. Afterward, one of the agents appeared in the center of the screen with straight gaze for a random time interval of 700–1000 ms. The agent then changed gaze direction either looking to the left or the right, followed by the appearance of one of the two target letters either at the valid or invalid location after a stimulus onset asynchrony (SOA) of 500 ms. Agent and target remained on the screen until a response was given or a time-out of 1200 ms was reached, whichever came first. At the end of each trial, a blank screen was presented for an inter-trial interval (ITI) of 680 ms before the next trial started.

Each session of the experiment was composed of 340 trials total, with a block of 20 practice trials preceding two experimental blocks of 160 trials each (one block per agent). The order in which the blocks were presented was counterbalanced across participants. Gaze direction (left, right), target side (left, right), target identity (F, T) and agent were selected pseudo-randomly and every combination appeared with equal frequency. Gaze validity was calculated based on the combination of gaze direction and target direction: on valid trials, the target appeared where the face was looking, while on invalid trials the target appeared opposite of where the face was looking. In the random condition, valid and invalid trials appeared with equal frequencies (i.e., 80 valid trials and 80 invalid trials per agent), whereas in the reliable condition, 80% of the trials were valid and 20% invalid (i.e., 128 valid trials and 32 invalid trials per agent); agent reliability was manipulated between participants. At the beginning and the end of each agent block, participants were asked to rate the agent’s capability of having a mind. For this purpose, the image of the respective agent was presented with a eight-point Likert scale presented underneath and participants were instructed to type in the number rating they wanted to assign to the agent into a free response box on the screen. No information about the actual reliability of the agents was disclosed at any time during the experiment.

### Analysis

Data was analyzed using R 3.2.4. Misses and incorrect responses, as well as data from participants with an accuracy rate below of 90% were removed prior to analyses (27% of trials). The data was analyzed with regard to the combined effect of appearance and behavior on (a) social-cognitive performance as measured in gaze-cueing effects and (b) agent ratings as measured in the degree to which mind was attributed to the agents. Gaze-cueing effects were calculated by subtracting the average reaction time for valid trials from the average reaction time for invalid trials (per participant, for agent and reliability conditions separately), and subjected to a 2 × 2 ANOVA with the within-factor Appearance (robot-like vs. human-like) and the between-factor Behavior (random vs. reliable). The more positive the difference score, the more strongly participants followed the gaze of the agent.

To examine how exposure to different appearances and behaviors changed the participants’ attitudes toward the agents, we calculated three mixed 2 × 2 ANOVAs with the within-factor Appearance (robot- vs. human-like) and the between-factor Behavior (random vs. reliable), and pre-interaction ratings, post-interaction ratings and difference scores between pre- and post-interaction ratings as dependent variables. A positive difference score between pre- and post-ratings reflects an increase in mind ratings after completing the gaze-cueing task (i.e., agent is perceived as more mindful after the interaction), while a negative difference score reflects a decrease in mind ratings after completing the task (i.e., agent is perceived as less mindful after the interaction). The higher the agent ratings at the pre- and post-interaction stage, the more willing participants were to ascribe mind to the gazer.

## Results

**FIGURE 3:**
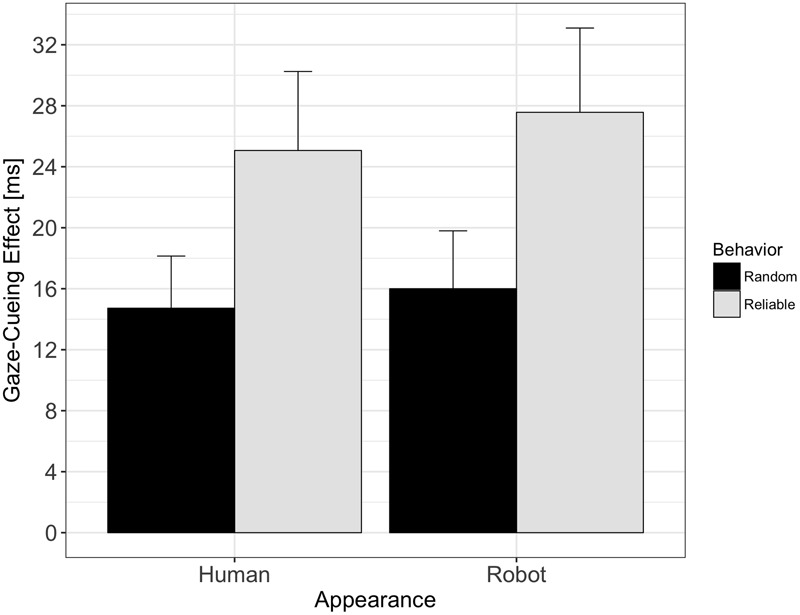
Gaze-cueing effects as a function of appearance and behavior: reliable agents induced significantly larger gaze-cueing effects than agents showing random behavior, independent of appearance. There was neither a significant main effect of appearance on gaze-cueing effects, nor was there a significant interaction effect between appearance and behavior.

The results of the analysis of the *gaze-cueing* data are shown in **Figure 3**. The 2 × 2 ANOVA revealed a main effect of Behavior [*F*(1,61) = 5.33, *p* = 0.024, $\eta_{p}^{2}$ = 0.04], with larger cueing effects for reliable versus random gaze behavior (26.4 ms vs. 15.4 ms). The main effect of Appearance was not significant [*F*(1,61) = 0.18, *p* = 0.67, $\eta_{p}^{2}$ = 0.001], suggesting that the gaze of the human-like agent was not followed more strongly than the gaze of the robot-like agent. The interaction effect between Appearance and Behavior was also not significant [*F*(1,61) = 0.02, *p* = 0.89, $\eta_{p}^{2}$ < 0.001], suggesting that the reliability with which the agent indicated target location influenced gaze following to the same degree for the human- and the robot-like gazer.

**FIGURE 4:**
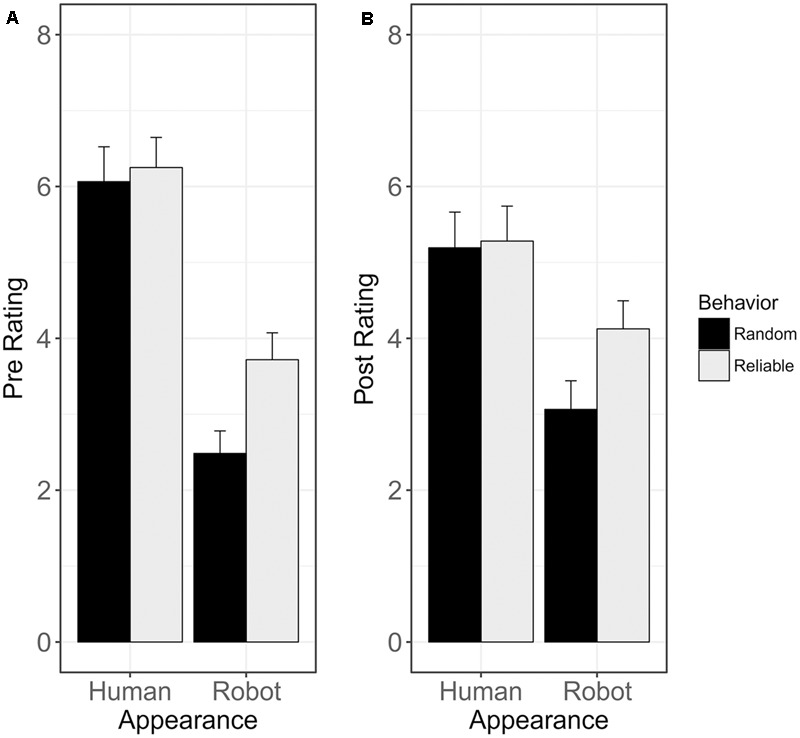
Mind judgments before and after gaze following: agent appearance affected mind ratings both before interacting with the agents during the gaze following task **(A)** and afterward **(B)**, with higher agent ratings for the human than for the robot agent. The reliability with which agents cued the target location did not have an effect on agent ratings, neither at the pre-interaction stage (i.e., no baseline difference in agent ratings between participants in the reliable and the random condition), nor at the post-interaction stage (i.e., knowing about the reliability of the agents did not influence the degree to which mind was ascribed to them). The interaction effect was not significant for either of the two rating times.

**FIGURE 5:**
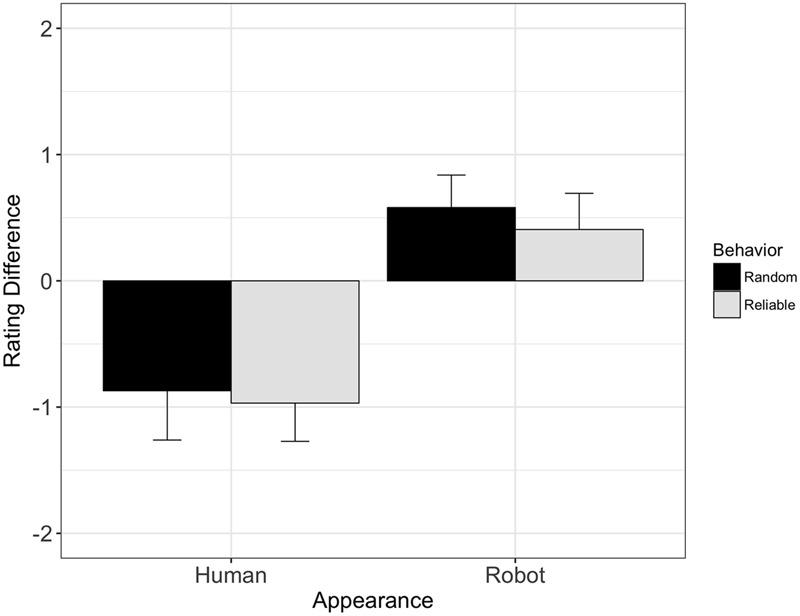
Change in mind judgments from pre- to post-interaction: mind ratings for the human-like agent decreased significantly during gaze following, while mind ratings for the robot-like agent increased during gaze following. Interestingly, this effect is independent of the reliability with which the agents predicted the target location during gaze following.

The results of the analysis of the *mind judgments* are shown in **Figures 4, 5**. The 2 × 2 ANOVA of the pre-interaction ratings revealed a significant main effect of Appearance [*F*(1,61) = 116.07, *p* < 0.001, $\eta_{p}^{2}$ = 0.34], with higher agent ratings for the human- than the robot-like agent (6.18 vs. 3.14). Neither the main effect of Behavior [*F*(1,61) = 2.39, *p* = 0.12, $\eta_{p}^{2}$ = 0.02], nor the interaction effect of Appearance and Behavior were significant [*F*(1,61) = 3.43, *p* = 0.06, $\eta_{p}^{2}$ = 0.01], indicating that there was no difference in the degree to which mind was attributed to the agents between reliability conditions prior to gaze following; see **Figure 4A**. The 2 × 2 ANOVA at the post-interaction stage showed a significant main effect of Appearance [*F*(1,61) = 38.95, *p* < 0.001, $\eta_{p}^{2}$ = 0.1], with higher ratings for the human- than the robot-like agent (5.29 vs. 3.63). Neither the main effect of Behavior [*F*(1,61) = 1.14, *p* = 0.28, $\eta_{p}^{2}$ = 0.01], nor the interaction effect of Appearance and Behavior [*F*(1,61) = 3.44, *p* = 0.06, $\eta_{p}^{2}$ = 0.01] were significant, showing that the agents’ reliability during gaze following did not influence how much mind was attributed toward them; see **Figure 4B**. The effect of Appearance on post-ratings was further modulated by participant gender with significantly lower ratings for the human-like agent by male participants than female participants [*F*(1,57) = 4.02, *p* = 0.04, $\eta_{p}^{2}$ = 0.05].

The 2 × 2 ANOVA of the difference scores between pre- and post-interaction ratings revealed a significant main effect of Appearance [*F*(1,61) = 25.13, *p* < 0.001, $\eta_{p}^{2}$ = 0.14], with a decrease in mind ratings for the human-like agent (Δ_80%_ = -1), and a slight increase in mind ratings for the robot-like agent (Δ_20%_ = +0.5). Neither the main effect of Behavior [*F*(1,61) = 0.16, *p* = 0.69, $\eta_{p}^{2}$ < 0.01], nor the interaction effect of Appearance and Behavior were significant [*F*(1,61) = 0.02, *p* = 0.89, $\eta_{p}^{2}$ < 0.01], showing that the agents’ behavior during gaze following did not affect how their mind status was rated; see **Figure 5**. The effect of appearance on changes in ratings from the pre- to post-interaction stage was further modulated by participant gender, with a significantly more negative change in ratings for the human-like agent for male than female participants [*F*(1,57) *=* 6.6, *p* = 0.01, $\eta_{p}^{2}$ = 6.26].

## Discussion

The goal of the current experiment was to examine whether appearance and behavior interact in their ability to trigger mind perception to non-human agents, and if so, how congruent (human-like/reliable vs. robot-like/random) versus incongruent (human-like/random vs. robot-like/reliable) combinations of these triggers affect social-cognitive performance (i.e., gaze following) and agent ratings (i.e., do you think the agent has a mind?). Based on previous studies, reliable gaze behavior and human-like appearance were expected to *increase* the likelihood that mind is perceived in artificial agents, while random gaze behavior and robot-like appearance was expected to *decrease* the likelihood for mind perception. To investigate whether and how these two triggers for mind perception interact, appearance and behavior were both manipulated within a gaze following paradigm, where either a human- or robot-like agent reliably or randomly cued the location of an upcoming target. If mind perception played a role for social-cognitive performance, gaze following should be stronger in conditions where mind was likely to be attributed to the gazer (i.e., human-like appearance, reliable behavior) compared to conditions where mind attribution was not likely (i.e., robot-like appearance, random behavior). Likewise, if appearance and behavior affected how agents were rated, more mind status should be attributed to them in conditions where mind perception was likely compared to conditions where it was unlikely.

The results show that agent behavior but not appearance affected gaze following, while agent appearance but not behavior affected mind judgments: gaze was followed more strongly in conditions where the agents showed reliable versus random gaze behavior, but this perception of reliability did not affect how much mind was ascribed to the agents after gaze following. In contrast, agent appearance did not have an impact on how strongly agent gaze was followed, but exclusively influenced mind attribution to the agents. Importantly, the positive effect of human appearance on mind ratings was observable both before and after participants interacted with the agent images during gaze following, and was not modulated by the reliability with which the agents cued an upcoming target location. Interestingly, however, the observed positivity bias caused by human-like appearance at first encounter seemed to fade over time (i.e., mind ratings for the human-like agent decreased between pre- and post-testing), while mind judgments for the robot-like agent increased from pre- to post-interaction ratings.

The observation that appearance and behavior influence how we interact with non-human agents is in line with previous reports showing that the two variables affect agent ratings (Looser and Wheatley, 2010; Waytz et al., 2010; Hackel et al., 2014; Martini et al., 2016), and performance (Kiesler et al., 2008; Morewedge, 2009; Süßenbach and Schönbrodt, 2014; Wiese et al., 2014; Mandell et al., 2015). Surprisingly, however, the current study shows that appearance and behavior differ significantly in their capacity to modulate performance versus mind judgments, with appearance having a stronger impact on agent ratings and behavior having a stronger impact on performance. This finding can be interpreted in two ways: first, it might indicate that judging one’s mind status is a qualitative rather than quantitative process, where agents either get mind status or no mind status ascribed, but nothing in between. If that were to be the case, it is possible that participants base their decision of whether an agent has a mind on just one mind trigger and ignore dissonant information from additional triggers to reduce potential cognitive conflicts. This interpretation is in line with previous studies showing that mind perception follows a qualitative pattern (i.e., significant increase in mind perception only after a certain threshold is passed; Cheetham et al., 2014; Hackel et al., 2014; Martini et al., 2016), and that conflicting information as to whether an agent has a mind or not has the potential to induce a cognitive conflict (Mandell et al., 2017; Weis and Wiese, 2017). Alternatively, the results could also indicate that mind perception is not a unified process that affects performance and attitudes in human–robot interaction in the same way, but instead that behavioral cues matter more in situations when participants actively interact with a robot agent, while physical cues have a stronger weight when making judgments about specific agent traits outside an interactive scenario. If that were to be the case, social roboticists would have to accentuate a robot’s perceived intentionality via behavioral cues when the robot’s main purpose is to engage in joint actions with human partners, as opposed to via physical cues when the focus of the interaction is on the robot’s personality.

Another unexpected observation in the current experiment was that mind ratings for the human-like agent decreased over time, while ratings for the robot-like agent slightly increased over time (both independent of reliability). With regard to the increase in ratings for the robot-like agent, it is possible that its mechanistic appearance might have primed participants to expect it to behave like a machine, incapable of engaging in social interactions. When they then experienced it sending social signals during the gaze following task, participants might have been positively surprised by the agent’s socialness, which in turn might have led to an increase in mind ratings. With regard to the decrease in ratings for the humanoid agent, it is possible that participants perceived a mismatch between its human-like appearance on the one hand and its mechanistic eye-movements on the other hand, with potentially negative effects on mind ratings. In gaze following paradigms, the impression of eye movements is caused by first presenting an agent looking straight and then, after a predefined time interval, the same agent looking to the side. Although this manipulation is effective in inducing shifts of attention to the gazed-at location, the eye movements usually do not match the biological motion patterns prototypically seen in human gazers. Since humans are quite sensitive to distinguishing biological motion from non-biological motion patterns (MacDorman and Ishiguro, 2006; Kätsyri et al., 2015; Wykowska et al., 2017), it is possible that perceiving a mismatch between human appearance and non-human motion might have triggered feelings of discomfort (MacDorman and Ishiguro, 2006; Saygin et al., 2012; Kätsyri et al., 2015), and therefore led to a decrease in mind ratings for the humanoid agent. The change in ratings from pre- to post-interaction was also more pronounced in male than in female participants, pointing at potential gender differences in perceiving mind in non-human agents.

The findings have several implications for the role perceptions of intentionality play in human–robot interaction. First, and foremost, the current study shows that expecting a robot agent to behave like an intentional being modulated attitudes and performance in human–robot interaction, and designing robots that trigger mind perception should therefore be an important goal to social roboticists. Second, although previous research has identified physical and behavioral factors that trigger mind perception in isolation, it seems like these triggers do not modulate attitudes and performance in human–robot interaction to the same extent. Rather, it seems that human-like behavior has a stronger impact on performance in human–robot interaction, while human-like appearance matters more when rating an agent regarding stable traits, such as ‘having a mind.’ Third, the current experiment shows that although human appearance has a positive effect on attitudes at first encounter, its effect seems to be short-lived and have detrimental consequences on human–robot interaction if the positive expectation caused by a robot’s appearance (i.e., agent behaves like a human) is not met by its actual behavior (i.e., agent does not behave human-like).

There are some limitations related to the current experiment. First, it is not clear to what extent the gender of the participant and the gender of the gazer (i.e., only a white male face was used as a basis for the morphed stimuli) had an influence on the reported results. While gaze-cueing effects and ratings at the pre-interaction stage were not influenced by participant gender, the post-interaction ratings and, in consequence, the changes in ratings over time, were modulated by participant gender, with a stronger decline in agent ratings for the human-like agent for male than female participants. Whether this effect is due to gender differences in mind perception or due to systematic biases of the current experimental setup cannot be determined based on the current data. Similarly, it is unclear whether using a wider range of gazing stimuli (i.e., different gender, age, ethnic background) would change the pattern of results reported in this paper. While it is common sense to control for perceptual features of the gazer by just using one gazing stimulus (i.e., of a particular gender, age and ethnic background) in gaze-cueing experiments (e.g., Hori et al., 2005; Bonifacci et al., 2008; Wiese et al., 2012; Wykowska et al., 2014; Graham et al., 2010), it cannot be ruled out completely that diversifying the features of the gazer might change the effects on mind ratings and gaze following reported in the current experiment. Second, we cannot fully rule out that changes in mind ratings from pre- to post-interaction are not simply due to a pragmatic effect, related to the fact that participants had to answer the same question (i.e., “Do you think the agent has a mind?”) twice, potentially suggesting to participants that the in-between manipulation was supposed to change their initial response. While this explanation is certainly possible, we do not believe that it is very likely, since asking the same question twice influenced participant answers differently for the human-like agent and the robot-like agent, with a decrease in ratings for the former and an increase in ratings for the latter. If asking the mind-rating question twice systematically impacted mind ratings in the current experiment, we would expect to see a similar effect for both the human-like agent and the robot-like agent. Since ratings do not change in the same way in both conditions, we believe that the observed changes in ratings are unlikely the result of a pragmatic effect. Third, asking participants whether they think an agent has a mind, might not actually measure *perceptions* of mind, but rather *judgments* of mind, that is: the reported ratings might not reflect the degree to which participants thought the depicted agents actually had minds, but rather how similar they thought they were to agents with mind (i.e., humans). While this limitation does not affect the general observation that mind ratings are affected by agent appearance, it might overestimate the degree to which participants actually see non-human agents as having a mind. Future studies need to address this issue by being more specific about whether they investigate mind perception or mind judgments.

## Author Contributions

AA and EW contributed to the conception and design of the study. AA collected and analyzed the data. AA and EW contributed to the interpretation of the findings of the study. AA and EW drafted the manuscript and revised it. EW provided the final approval to publish the manuscript. AA and EW agree to be responsible for ensuring that questions related to the accuracy or integrity of the study would be resolved.

## Conflict of Interest Statement

The authors declare that the research was conducted in the absence of any commercial or financial relationships that could be construed as a potential conflict of interest.
